# One-stage Surgical Treatment of Simultaneous Osteotomy and Asymmetric Lengthening on Short Femur with Severe Deformity of Genu Valgus

**DOI:** 10.1038/s41598-019-45157-4

**Published:** 2019-06-13

**Authors:** Hui-Fa Xu, Chao Xu, Jia Sha, Ya-Bo Yan, Chao Li, Zhi-Chen Liu, Lu-Yu Huang, Wei Lei

**Affiliations:** 1 Department of Orthopedics, Xijing Hospital, Air Force Military Medical University, Xi’an, 710032 China; 2 Department of Orthopedics, Ningxia People’s Hospital, YinChuan, 750021 China

**Keywords:** Paediatric research, Paediatric research

## Abstract

This study aimed to discuss the effects of one stage surgical treatment by simultaneous osteotomy and asymmetric lengthening by Ilizarov external fixator on short femur with severe deformity of genu valgus. A total of 12 cases with unilateral deformities treated by simultaneous osteotomy and Ilizarov asymmetric lengthening on short femur with severe deformity of genu valgus were retrospectively analyzed from January 2006 to April 2015. The affected limbs were 2.5–11 cm (5.2 cm on average) short, the femorotibial angle was 135°–158° (146.3° on average), and the ankle interval was 15–43 cm (24.7 cm on average). The Paley method was used to determine the osteotomy plane (distal femur) of genu valgus. According to this standard, the bone union results were as follows: 11 had excellent and 1 had good, where 7 patients had excellent and 5 had good functional outcomes. One stage surgical treatment by simultaneous osteotomy and asymmetric lengthening on short femur with severe deformity of genu valgus was considered to be an effective and reliable method with better osteotomy union, less trauma and fewer complications.

## Introduction

Epiphyseal injuries are caused due to trauma, osteomyelitis, tumors, developmental malformations, radiation damage, and iatrogenic injuries. Of these, trauma remains to be the most common cause^1,2^, and about 1/3 children (with 42% boys and 27% girls) of 0–13 year olds experience fractures due to trauma or playing sports^3^. The fracture occurrence rate has been decreased by 9% since 2013^4^. Approximately 18% of the fractures are associated with growth plate and its damage can result in angulation deformity or inhibited growth^5^. The cartilage at the injury region is replaced by bone, subsequently forming the bone bridge or the bony bar. The injury of distal femur epiphyses can cause 35% premature closure of epiphyseal plate and bony bridge formation^6^. The short femur with severe deformity of genu valgus usually occurs due to epiphyseal injury and bone bridge formation, which is treated by bone bridge resection during the early stages^7,8^. Knee varus, knee valgus, anteflexion, recurvatum and other short femur deformities, such as genu varus and valgus with short femur of different levels till the closure of the epiphyses, are caused due to no resection or partly resection of the bone bridge or the bone bar. It not only affects the quality of life of the patients’, but also causes severe psychological burden. However, clinical treatment of these types remains to be difficult. Thus, supracondylar femur osteotomy and 8-plate hemi epiphysiodesis are used to treat severe articulation genu angulation deformities during the later periods in the first stage^9–12^, and bone lengthening is performed in the second stage^13,14^. This therapy involves complicated process of long treatment cycle, high expenses and severe trauma, and the short femur deformity is regarded to be more severe if the angulation deformity is treated. So, the hospital stay, the expenses and the complications associated with it are increased during bone lengthening period. Hence, in this study, simultaneous osteotomy of distal femur supracondylar was performed, and the different extended lengths of the medial and the lateral extension poles were accurately calculated by using the formula. Simultaneous osteotomy and asymmetric lengthening caused less damage to the soft tissues with maximal advantages of maintaining the bone mass, reducing the chances of nerve and blood vessel injuries, and achieving functional training earlier by a surgery. These in turn showed that it is an effective way for the treatment of short femur with severe deformity of genu valgus.

## Methods

### Patients

This study was approved by the Ethics Committee of the Air Force Military Medical University. All experiments were conducted in accordance with the Declaration of Helsinki. Written informed consent was obtained from all participants, their parents and/or legal guardians before study initiation. Twelve cases treated by simultaneous osteotomy and Ilizarov asymmetric lengthening on short femur with severe deformity of genu valgus from September 2009 to April 2015 were retrospectively analyzed. The patients with torsional deformity and sagittal plane angulation deformity were excluded from the study. There were 4 males and 8 females, in which 7 cases were due to traffic injuries, 2 by high falling, 1 by heavy crashing object, 1 by osteomyelitis epiphyseal injury, and 1 by iatrogenic injury. The patients age ranged from 13 to 21 years (15.9 years on average), had unilateral deformities of short femur of 2.5–11 cm (5.24 cm on average), the femorotibial angle of 135°–158° (146.3° on average), and the ankle interval of 15–43 cm (24.7 cm on average).

### Pre-operation preparation and design

**Figure 1:**
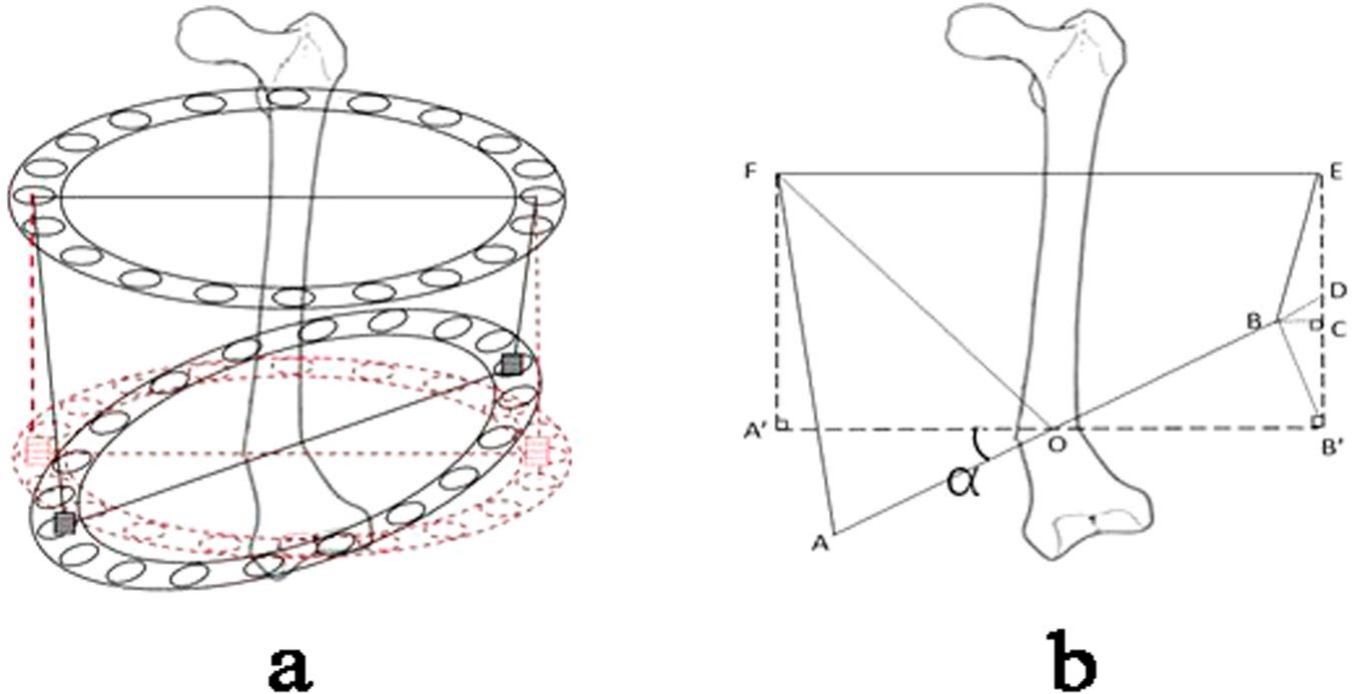
(**a**) Pre-adjustment scheme of external fixator after operation. (**b**) Simplified geometry model. AF, indicates the medial extension pole length; BE, the lateral extension pole length; R = ($\frac{1}{2}$ EF), the semi-diameter of the ring; α, the proposed corrective remediable femur valgus angle.

Full-length anteroposterior X-ray of both lower extremities in erect position, and full-length anteroposterior and lateral X-ray on bilateral femur and tibia were performed to measure the length of bilateral femur and tibia, the femoral tibial angle (FTA), tibial angle, femoral angle, the tibiofemoral articular surface tangent line intersecting angle and the lateral tibiofemeral interval. The partial bone morphology changes of deformity sources, the limb alignment changes and the motion of the joint were also analyzed. Through Paley method, osteotomy plane and the center of rotation angulation (CORA) on femoral valgus were confirmed to determine the extended length and orthopedic angle. According to Paley’s multiplier prediction analysis, the discrepancy levels of limb length after children’s skeletal maturation were calculated to confirm the lengthening extension after surgery. The extended length of the external fixator’s medial and lateral extension poles was accurately calculated and the formula was given below (Fig. 1 and Formula 1 and Formula 2):

The geometric formula is as follow (see the derivation process, Supplemental Content):

1 $${\rm{Y}}={\rm{L}}+{\rm{k}}[{\rm{R}}\cdot \,\tan \,{\rm{\alpha }}+\sqrt{{{\rm{BE}}}^{2}-{(1-\cos {\rm{\alpha }})}^{2}{{\rm{R}}}^{2}}-{\rm{R}}\cdot \,\sin \,{\rm{\alpha }}\cdot (1/\,\cos \,{\rm{\alpha }}-1)-{\rm{BE}}]$$

2 $${\rm{X}}={\rm{L}}-{\rm{k}}^{\prime} \{{\rm{AF}}-[{\rm{R}}\cdot \,\tan \,{\rm{\alpha }}+\sqrt{{{\rm{BE}}}^{2}-{(1-\cos {\rm{\alpha }})}^{2}{{\rm{R}}}^{2}}-{\rm{R}}\cdot \,\sin \,{\rm{\alpha }}\cdot (\frac{1}{\cos \,{\rm{\alpha }}-1})]\}$$

Formula 1 and Formula 2: AF, indicates the medial extension pole length; BE, the lateral extension pole length; ${\rm{R}}=(\tfrac{1}{2}{\rm{EF}})$, the semi-diameter of the ring; α, the proposed corrective remediable femur valgus angle; L, the extended length of the affected limb; Y the extended length of lateral extension pole; and X the extended length of medial extension pole. However, according to our pervious clinical observation, there is a certain error, and the adjustment using the formula for the correction of the angulation deformity is not thorough. Thus, the formula was slightly adjusted, and the adjustment coefficient K and K’ are brought in. The adjustment coefficients are set as K = 1.09 and K′ = 0.9 according to the clinical experiences.

### Surgical methods

The patient was placed in supine position on an X-ray see-through operation bed, and requested the affected limb to elevate to 30°. The lumbar anesthesia or general anesthesia was then applied, and the general skin preparation and draping were performed. The osteotomy plane was selected according to the pre-operation design. Two Steinmann pins (2.5 mm in diameter) were placed perpendicularly to the backbone from the osteotomy plane and 2 Steinmann pins (2.5 mm in diameter) were crossed into the distal femur parallel to the articular surface. Ilizarov external fixator was placed in order to position the femur in the center or the external fixator ring. One Steinmann pin (2.5 mm in diameter) was placed on the “C” shape ring from the proximal femur, and 1 more Steinmann pin or 2 Shan’s pins were placed for fixation from the proximal femur. Subcutaneous and deep fascia of the skin of 5 cm was incised from the middle and distal femur to enter from the posterior vastus lateralis muscle to expose the pre-selected osteotomy plane. Using fluoroscopy, the osteotomy was applied parallel to the articular surface of the distal femur. The external fixator was checked for stableness, and then for hemostasis, washed, closed the wound and applied sterile gauze.

### Postoperative management

Anti-infection detumescence and symptomatic support were provided in all patients. Functional training was conducted for 2 days after the surgery with pin hole care. The external fixator was lengthened asymmetrically 1 week after the surgery. The length of the medial and lateral extension pole was calculated and the overall lengthening days were determined by the shortened length of the femur L (to guarantee the femur was lengthened 1 mm per day). The lengthening times were divided into 4 and then the extension length per day was calculated. The articulatio genus underwent exercise, and the end-feel, blood supply and progression in the activity were observed. The X-ray was reviewed regularly, and the lengthening speed was adjusted according to the growth and calcification of the callus. The callus growth condition was observed to check if the lengthening expectation could be met. The external fixator was taken off until the continuous bone cortex was seen from the femur extension point (the 4 layers of the bone cortex were seen from anteroposterior and lateral film). Orthosis was applied for ground activity for 1–2 months as a protection. The femur length discrepancy and the articulatio genus mobility were measured and the external fixation index was calculated by using the formula: EFI: the fixator carrier duration (month)/extension length (cm)^15^.

### Evaluation indicators

All patients were evaluated by bone nonunion healing and the functional recovery methods were established by Paley^16^.

### Complications

The complications during the treatment periods were divided into minor, major and permanent complications. Minor complications could be managed without any operation, the major complications could be treated by surgery, and the permanent complications persisted till the end of the therapy^17^.

## Results

All the cases were followed-up for 12–60 months (32.8 months on average). The femur was lengthened by 2.3–10.2 cm (5.4 cm on average), the femoraotibial angle was 171°–177° (173.4° on average), and the affected limb was shortened by −1.0–1.1 cm (0.17 cm on average) postoperatively. The treatment of genu valgus and short femur remained satisfying, and the patients were able to walk without pain and showed no bone nonunion and peroneal nerve injuries postoperatively. Ten cases had extension and flexion restriction [extension (0°) – flexion (15°–90°)], and had better condition after undergoing physiotherapy [the extension (0°) – flexion (80°–125°)] till the last follow-up. According to Paley, the standard synostosis results showed 11 with excellent and 1 good, and the functional outcomes showed 7 as excellent and 5 good (Table 1).

**Table 1:** Detailed data of 12 children.

case	Age (Y)	gender	side	Posttraumatic time (Y)	Pre TFA/aLDFA	Shortening length	intermalleolar distance	Adjustment time (D)/External fixation time (M)	Follow up (M)	end of follow up TFA/aLDFA	Shortening length	intermalleolar distance	range of motion of the knee joint	accordion technique time
1	13	F	L	10	151/50°	4.5 cm	17	50/9	41	173°/78°	−0.6 cm	3 cm	0°/100°	
2	11	F	R	7	152°/49°	4 cm	15.5	53/8.5	38	174°/73°	−1 cm	3.6 cm	0°/115°	
3	16	M	L	14	138°/35°	8 cm	43	100/15.5	12	175°/73°	0.3	1.5 cm	0°/80°	1 week
4	20	F	L	19	138°/43°	3 cm	21	32/8.5	48	175°/81°	0.2 cm	1 cm	0°/105°	
5	12	F	R	6	148°/50°	4.3 cm	15	51/8.5	50	174°/79°	−1 cm	2.3 cm	0°/120°	
6	14	F	L	5	151°/55°	3.5 cm	16	37/6.5	60	177°/80°	−0.5 cm	3 cm	0°/125°	
7	21	M	R	11	155°/59°	3.3 cm	24	33/6	36	172°/83°	0 cm	1 cm	0°/100°	
8	16	M	L	8	144°/55°	5 cm	28	55/11	30	173°/83°	−0.5 cm	2.5 cm	0°/110°	
9	19	F	L	12	135°/41°	6.8 cm	36	85/12	24	174°/85°	1.1 cm	2.8 cm	0°/100°	1 week
10	14	F	R	6	147°/59°	7 cm	29	85/14	12	171°/84°	−1 cm	5 cm	0°/105°	
11	20	M	R	6	158°/63°	2.5 cm	15	25/8	24	173°/80°	0.2 cm	3.3 cm	0°/95°	
12	15	F	R	13	135°/41°	11 cm	37	130/20	18	170°/77°	0.8 cm	5.7 cm	0°/85°	2 week

**Figure 2:**
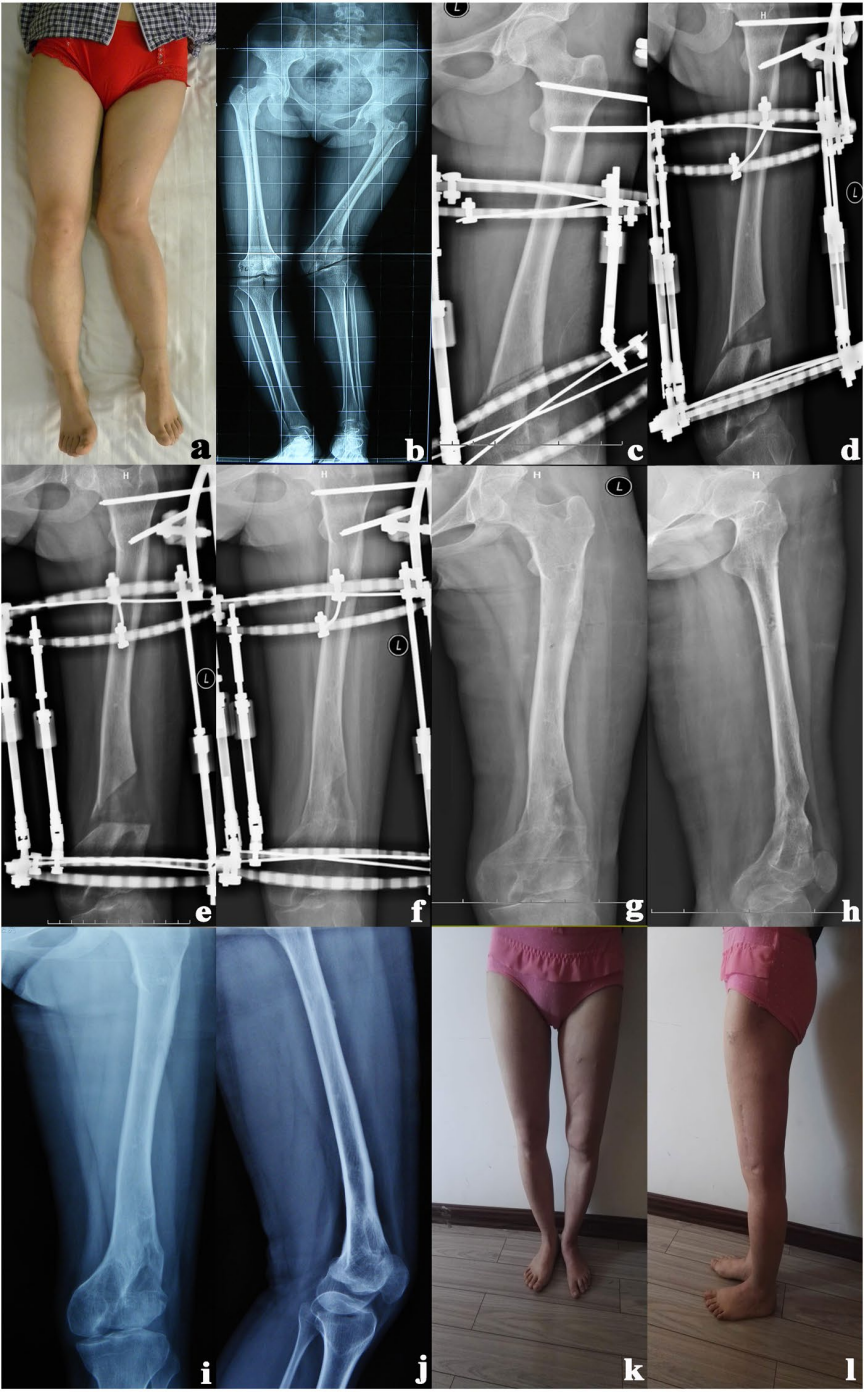
A 20-year-old female patient had short femur with severe genu valgus. (**a**) Short femur with severe genu valgus of the left lower limbs before operation. (**b**) Preoperative X-ray scan: the left femur was 30 mm short, FTA:138°, aLDFA:43°. (**c**) The osteotomy plane should be parallel to the distal femoral articular surface. (**d**) extended for 14 days and the lateral extension pole was adjusted to 28 mm. (**e**) Extended for 32 days and the lateral extension pole was adjusted to 64 mm. (**f**) The callus of the extended part was mature after lengthening for 8.5 months. (**g**,**h**) The external fixator was removed. (**i**,**j**) The osteotomy showed no loss. k, l: the anterior and lateral force lines of the left lower limb were good postoperatively after 4 years.

The minor complications observed in 10 cases included Checketts and Otterburn external fixator’s level 1–2 screw path infection, which were cured after Anerdian disinfection and oral antibiotic administration. Major complications were observed in 2 cases suffering from level 4 screw path infection, which were cured through screw path expansion and clean-up by reoperation as well as intravenous antibiotic therapy. In 1 case, one locking pin showed breakage, which was replaced afterwards. Real complications occurred in 6 cases, which included knee osteoarthritis and knee pain after long-distance walk. The occurrence of these complications was 1.6 type/1 case. A typical case was shown in Fig. 2.

### Typical case

A 20-year-old girl with severe deformities for over 19 years underwent semi-diameter measurements of the Ring R = 97 mm, the medial extension pole length AF = 205 mm, the lateral extension pole length BE = 135 mm, the femorotibial angle 138° (the proposed corrective remediable femur valgus angle α = 36°), and the extended length of the affected limb L = 30 mm. According to the calculation, the extended length of the medial extension pole was 66 mm, and the extended length of lateral extension pole was −0.4 mm, indicating that the shortened length was 0.4 mm. The extension therapy for the external fixator of the affected limb was conducted by applying the external fixator asymmetrically 1 week after the surgery, and the 2 lateral extension poles were lengthened 2 mm every day, while the 2 medial extension poles were unadjustable. During the extension period, X-ray was taken to observe the adjustment of the angulation and the new bone growth condition of the osteotomy part. The results showed that the extension was stopped when the lower limbs mechanical axis and the length were proved to be recovered by X- ray scanning after 32 days of extension. After that, reexamination by using X-ray was performed every month. The external fixator was removed when the callus appeared satisfying by X-ray scanning 8.5 months later and good bone healing. Both the lower extremities were discovered with equal length (the femur was short for 0.2 cm), and the femorotibial angle was 175° 4 years after surgery. The adjustment of angulation deformity and the shortening deformity showed satisfactory results, with recovered lower limb mechanical axis, walking without pain, no bone union and peroneal nerve injury discovered on physical examination.

## Discussion

The above treatment demonstrated good therapeutic effects in all the 12 cases. The merits of this study are discussed in the following paragraphs.

Firstly, the bone mass was maintained maximally. The staged operations were applied in traditional therapies, wherein osteotomy was applied to adjust the angular deformity, and the limb lengthening was applied after osteotomy healing for those limbs with length discrepancy^11,13^ or the 8-plate was used to block the epiphyseal growth^12^. The bone mass loss of the short femur and the blocking of the epiphyseal growth occurred due to the above discussed methods, and the femoral growth was delayed, bringing more difficulties and prolonging the fixation time during the second stage of femoral lengthening.

Secondly, the formula for calculating the extended length and speed of the medial and lateral extension pole was accurately given for the first time, providing the technical support for the manipulation as well as enriched the theory of Ilizarov distraction osteogenesis. The short femur was extended to the expected length and the angular deformity was adjusted to the expected angulation, and the possible nerve and blood vessel injury was avoided during distraction lengthening. Since the nerves, blood vessels, periosteum and other tissues could not tolerate the fast-speed distraction lengthening, it was better to carry out gradual distraction lengthening for biological adaptation. Therefore, the distraction speed remains the key point during the procedure. Nerves, blood vessels, periosteum and other tissues could be gradually lengthened through slow distraction, and induce synchronous growth under slow and limited distraction. Animal experiments showed that gradual lengthening could help tubular membranes and blood-nerve barrier to maintain integrity, and the blood circulation was scarcely influenced, contributing to the stability of microcirculation and intraneural microenvironment. However, prolonged external fixation time by slow lengthening may take additional time for therapy afterwards. So, how to lengthen as fast as possible without compromising the nerve and blood vessels remains to be the key point in this research. According to the formula, the daily length was calculated and the lengthening was divided into 4 times, i.e., femur axis was lengthened 1 mm per day. The lengthening plan could be tailored according to patient’s age, bone condition, nutritional status, other factors, and X-ray scan 1 week before the proposed lengthening was required for appropriate adjustment.

Thirdly, the EFI was lower and the patients could have free activities as earlier. Compared with 1–2 months/cm in the previous reports^18,19^, the average callus index of these cases was 2.07 months/cm (1.60–3.48 months/cm), and was influenced by the type of disease, age, osteotomy site, surgical history, and other factors. According to Fischgrund J^15^, the longer the lengthening was, the smaller the index was. The EFI in these cases was slightly higher than that reported in the previous studies. This was because the indicator for removing the external fixator in the previous reports was 3 layers that of the bone cortex in the anteroposterior and lateral films, while the indicator in our study was continuous bone cortex at the femoral lateral lengthening part, and the 4 layers of continuous bone cortex were discovered, leading to increased fixation time. While the EFI was 1.64 (1.33–2.58) according to the femoral lateral lengthening, and by considering different indicators, which were still equivalent to that in the previous reports, which indicated that the fixation time was not prolonged by the adjustment of angulation deformity (Table 2).

**Table 2:** Detailed data of 12 children.

case	Age (Y)	gender	side	Extended length (cm)	corrective angle	Lateral extended length (cm)	Medial extended length (cm)	External fixation time (M)	EFI
1	13	F	L	5.1	23°	5.9	4.3	9	1.76
2	11	F	R	5	22°	5.7	4.3	8.5	1.70
3	16	M	L	7.7	36°	8.9	6.5	15.5	2.01
4	20	F	L	2.8	36°	4.1	1.5	8.5	3.04
5	12	F	R	5.3	26°	6.3	4.3	8.5	1.60
6	14	F	L	4	23°	4.9	3.1	6.5	1.63
7	21	M	R	3.3	19°	3.8	2.8	6	1.82
8	16	M	L	5.5	30°	6.5	4.5	11	2.0
9	19	F	L	5.7	39°	7.8	3.6	12	2.11
10	14	F	R	8	27°	8.9	7.1	14	1.75
11	20	M	R	2.3	16°	3.1	1.5	8	3.48
12	15	F	R	10.2	39°	11.8	8.6	20	1.96

The correct installation of the external fixator is the key factor for the success of the surgery. The external fixator should be secured and reliable, and otherwise, it is not good for bone regeneration and repair, as bone grafting is applied in 15% of patients caused due to nonunion or delayed union^20^. Firstly, the external fixator proximal ring should remain perpendicular to the femoral shaft, and the distal ring should remain parallel to the distal femoral articular surface. Secondly, to calculate the extended length of the extension pole, the femur should be in the center of the external ring. This was due to that the anatomical factors may cause errors in calculation. With the use of both animal experiments and clinical data, the corrective coefficients K and K’ were calculated by regression analysis. Thirdly, 4 extension poles are applied, in which the spaces between each extension pole are the same and the coronal and sagittal planes of the 4 extension poles should remain symmetrical.

According to Paley, the adjustment should maximally recover the mechanical axis of the lower limb, and the limbs should be recovered at the same time for the discrepancy of limbs. Meanwhile, the relations among CORA, angulation correction axis (ACA) and osteotomy plane should be identified^21^. When the osteotomy plane is located at the CORA level along with the osteotomy site as angulation correction axis, no osteotomy end displacement was observed after the correction. If the osteotomy plane was far from CORA, and was still with the CORA as angulation correction axis, the mechanical axis becomes normal, but the osteotomy ends showed displacement. Since the deformity is caused by epiphyseal injury, and the CORA of genu valgus was usually located at the epiphyseal plate or near to the epiphyseal plate, osteotomy was considered unsuitable at this place for the injury of epiphyseal plate for the second time. As the osteotomy plane usually moves upward towards the femoral supracondylar level, the osteotomy plane cannot be in the same plane. For the recovery of limb alignment, the ACA of the affected limb should go through the CORA point. Otherwise, the femoral distal and proximal axis cannot be in the same line after deformity correction. The osteotomy plane should be closer to the CORA plane for preventing the displacement of the osteotomy ends between each other.

The complications should also be prevented. Stent-tract infection, external fixator locking pin breaking, osteoarthritis and the other 16 types of complications are discovered, with 1.6 types/cases on an average. The stent-tract covers 100% complications, and is observed in all patients at various levels, but the change of the fixator locking pin and intravenous antibiotics are not required. According to the previous reports, the occurrence of stent-tract infection was 96.6% during the external fixator lengthening, and the occurrence of stent-tract infection caused by the change of the locking pin and intravenous antibiotics accounted for 5.8%^22^. Stent-tract infection is caused by many factors, and the reduction of the occurrence rate requires joint efforts of the doctors and patients. So, doctors should pay more attention to the following aspects. Firstly, the needle should be slowly inserted to prevent the bone tissue thermal necrosis. Secondly, the slice part of the tissue should be chosen to insert the needle. Thirdly, it is better to use smooth Kirschner wire or Steinmann pin, and if thread needles are required, the thread part should be kept in soft tissues totally. Fourthly, the external fixator needle should be debonded to reduce the pressure of the soft tissue for preventing high skin tension. Fifthly, the needle should be prevented from inserting into the soft tissue of higher mobility, and the movement of the needle should be reduced around the soft tissue. Sixthly, the pore diameter of the external fixator ring should be proper as it remains unstable if too big and the stent-tract infection can cause effusion due to small space between the ring and the skin. Seventhly, the area should be cleaned around the screw path with a disinfectant, and the prevention of the callus at the screw path remains the key point to prevent stent-tract infections. Eighthly, the doctors should communicate well with the patients and provide timely management of infections, which are the key points for the prevention of stent-tract infection. In our study, 6 patients had poor development of femoral lateral condyle and imbalance of distal and proximal femoral articular surface before the treatment. Although osteoarthritis was relieved after the conduction of short femur and limb alignment, it required joint replacement in future.

The external fixator should be dismantled in time. The imaging criterion for dismantling the external fixator after bone lengthening is that the continuous cortical bone can be observed at the lateral femur of the lengthened new bone. It is reported^23^ that the occurrence of the new bone bent and fracture accounted for 19% if based on the imaging criterion only. Besides the imaging criterion, other factors such as functional recovery, unscrewing the fixed end to reach motorization, and walking without pain should also be considered for dismantling the external fixator safely^24^. In this paper, we hypothesized that it was not easy to walk by wearing external fixator, and the factures are easily caused by stress concentration of the adjusted angulation. So, it is considered safe to dismantle the external fixator when the continuous cortical bone is discovered at the lateral femur, and the load bearing should be protected for 4–6 weeks after dismantling the external fixator.

Follow-up of the cases after surgery showed that stage-one surgical treatment by simultaneous osteotomy and asymmetric lengthening was an effective approach for the treatment of short femur with severe deformity of genu valgus, assisting in the reduction of treatment cycle as well as expenses. The logical preoperative design, the precise operation and the accurate calculation of the postoperative lengthening plan are the key factors for surgical success, and other important factors included the prevention and treatment of complications, the perioperative psychological comfort, early rehabilitation and physical therapy and other measures. Doctors, patients and guardians required a tight cooperation, and negligence of any measure could cause failure of the surgery.

The treatment of short femur with genu valgus in this manuscript showed improvement under local conditions in China, as most of the hospitals still chose staged surgeries, though Taylor Frame has been popular and proven effective in developed countries. The mathematical formula we calculated assisted us in achieving accurate simultaneous correction and reducing the number of operations. The osteotomy was uniform and the correction was performed at constant speed, which in turn can help for the even growth of the bone, thus reducing the time of external fixator. This method is an innovative and practical as the Taylor Frame is not widely used in developing countries. With China’s special national conditions, the use of Ilizarov fixator is considered as the main treatment method, and constant updation of the knowledge is warranted to improve and experience the perfection in the use of technology. Due to economic conditions and technical capabilities, surgery in two-stage remains very common in China. This article provided practical significance and new insights by guiding Chinese doctors to perform a one-stage operation, finally helping the patients with reduced pain, duration of hospitalization and surgical procedure costs. It is well known that Taylor Frame has been widely used for complicated cases with lower limb deformities in developed countries and has achieved good results^25–27^. In view of these results, we aimed to use Talor Frame and planned to use it in clinics soon. Intramedullary lengthening nail is expensive, and so it has not been applied in clinical practice in China. According to the literature^28^, femoral lengthening with femoral intramedullary lengthening nail achieved excellent functional results with fewer complications and greater patient satisfaction when compared with external fixator monorail system. With the development of technology and the reduction of costs, intramedullary lengthening nail may be one of the main developmental directions of limb lengthening and bone reconstruction.

## Supplementary information

Appendix for the formula derivation process
