# Supplementary material for: One-stage Surgical Treatment of Simultaneous Osteotomy and Asymmetric Lengthening on Short Femur with Severe Deformity of Genu Valgus
Source: Sci Rep. 2019 Jun 13;9:8602. doi: 10.1038/s41598-019-45157-4 (PMC6565623; doi:10.1038/s41598-019-45157-4)
Supplement: Supplementary file 1 — Appendix for the formula derivation process [file 41598_2019_45157_MOESM1_ESM.docx]

**Title:** One-stage Surgical Treatment of Simultaneous Osteotomy and Asymmetric Lengthening on Short Femur with Severe Deformity of Genu Valgus

**Authors:** Hui-Fa Xu, Chao Xu, Jia Sha, Ya-Bo Yan, Chao Li, Zhi-Chen Liu, Lu-Yu Huang, Wei Lei

The formula derivation process is as follows:


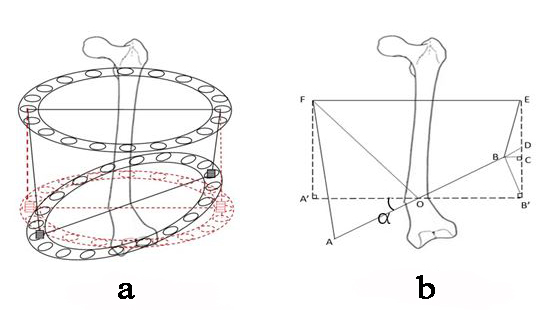


AF, indicates the medial extension pole length; BE, the lateral extension pole length; R=($\frac{1}{2}$EF), the semi-diameter of the ring; α, the proposed corrective remediable femur valgus angle; The inner screw length A’F and the outer screw length B’E are set for the correction of the angulation deformity. Therefore, the inner screw length should be shortened as X’=AF-A’F, and the outer screw length should be lengthened as Y’=B’E-BE to correct the angulation deformity.

Then,

B’D=$R\cdot\tan\alpha$

OD=$R/ \cos\alpha$

BD=OD-OB=$R/ \cos\alpha-R$

BC=$\mathrm{OB}'\cdot\mathrm{BD}/\mathrm{OD}$=$R\cdot R(1/{\cos\alpha}-1)/(R/cos\alpha)$=$(1-\cos\alpha)\cdot R$

CE=$\sqrt{\mathrm{BE}^{2}-\mathrm{BC}^{2}}$

B’E= B’D+DE= B’D+ CE-CD

CD=$\mathrm{BD}\cdot\sin\alpha$=$(OD-OB) \cdot\sin\alpha$= $(R/ \cos\alpha-R) \cdot\sin\alpha$

So, in order to correct the angulation deformity, the outer screw of the affected limb should be lengthened as

Y$'$ =B’E-BE= B’D+ CE-CD-BE

=$R\cdot\tan\alpha+\sqrt{\mathrm{BE}^{2}-\mathrm{BC}^{2}}- (R/ \cos\alpha-R) \cdot\sin\alpha-\mathrm{BE}$

=$R\cdot\tan\alpha+\sqrt{\mathrm{BE}^{2}-\mathrm{BC}^{2}}- (R/ \cos\alpha-R) \cdot\sin\alpha-\mathrm{BE}$

=$R \cdot\tan\alpha+\sqrt{\mathrm{BE}^{2}-\left( 1-\cos\alpha\right)^{2}R^{2}}-R \cdot\sin\alpha\cdot(1/\cos\alpha-1)-\mathrm{BE}$

so, in order to correct the angulation, the inner screw of the affected limb should be shortened as

X$'$= AF-A’F= AF –B’E

=$\mathrm{AF}-\left( R\cdot\tan\alpha+\sqrt{\mathrm{BE}^{2}-\mathrm{BC}^{2}}- (R/ \cos\alpha-R) \cdot\sin\alpha\right)$

=$\mathrm{AF}-\left[ R \cdot\tan\alpha+\sqrt{\mathrm{BE}^{2}-\left( 1-\cos\alpha\right)^{2}R^{2}}-R \cdot\sin\alpha\cdot(1/\cos\alpha-1) \right]$

For patients with femoral angulation and shortening deformity (shrinking L compared with the healthy side) at the same time, in addition to correcting the valgus deformity, the inner and outer screws need to be adjusted simultaneously to correct the femoral shortening deformity. Therefore, the inner screw is adjusted as L-X’ and the outer screw is lengthened as L+Y’

$$L+Y'=L+[R \cdot\tan\alpha+\sqrt{\mathrm{BE}^{2}-\left( 1-\cos\alpha\right)^{2}R^{2}}-R \cdot\sin\alpha\cdot(1/\cos\alpha-1)-BE]$$

$$L-X^{'}=L-\left\{ \mathrm{AF}-\left[ R \cdot\tan\alpha+\sqrt{\mathrm{BE}^{2}-\left( 1-\cos\alpha\right)^{2}R^{2}}-R \cdot\sin\alpha\cdot\left( \frac{1}{\cos\alpha-1} \right) \right] \right\}$$

However, according to our pervious clinical observation, there is a certain error, and the adjustment using the formula for the correction of the angulation deformity is not thorough. Thus, the formula was slightly adjusted, and the adjustment coefficient K and K’ are brought in. The adjustment coefficient are set as K=1.09 and K’=0.9 according to the clinical experiences.

$Y$=$L+k[R \cdot\tan\alpha+\sqrt{\mathrm{BE}^{2}-\left( 1-\cos\alpha\right)^{2}R^{2}}-R \cdot\sin\alpha\cdot(1/\cos\alpha-1)-BE]$(formula-1)

X=$L-k^{'}\{AF-\left[ R \cdot\tan\alpha+\sqrt{\mathrm{BE}^{2}-\left( 1-\cos\alpha\right)^{2}R^{2}}-R \cdot\sin\alpha\cdot\left( \frac{1}{\cos\alpha-1} \right) \right]\}$(formula-2)
